# Telling our own story in global health–experience from Nigeria

**DOI:** 10.1371/journal.pgph.0000735

**Published:** 2022-07-20

**Authors:** Chikwe Ihekweazu, Ifedayo Morayo Adetifa

**Affiliations:** 1 World Health Organization, Berlin, Germany; 2 Nigeria Centre for Disease Control, Abuja, Nigeria; PLOS Global Public Health and APHRC, KENYA

Much has been written about the inequalities that exist in the development and dissemination of scientific knowledge from low- and middle-income countries [LMICs] [1–3]. However, less is known and shared about the responsibility of governments and institutions in LMICs in turning this tide. While we agree with the notion that there are perverse incentive structures in the West that allow these inequalities to occur, our experience in Nigeria shows that there is a role for LMICs to play in addressing these.

The Nigeria Centre for Disease Control [NCDC] is Nigeria’s national public health agency [4]. Most national public health agencies focus on their core responsibilities around the preparedness, detection and response to infectious disease outbreaks and other public health threats [5]. However, in addition to these, developing new knowledge on the threats that we face is critical to improving the efficiency of these core responsibilities. In Nigeria, we have the opportunity that the Act establishing NCDC explicitly requires it “to conduct, collate, synthesize and disseminate public health research to inform policy and guidelines of diseases of public health importance…” [6]. If national public health agencies are not intentional in learning from their experiences with scientific rigour, then there is very little chance that we will make progress on response activities.

Throughout the year, Nigeria experiences and responds to various infectious disease outbreaks. The annual peak of Lassa fever cases is typically observed during the dry season between December and April [7]. During the rainy season which usually lasts from March to September, we see an increase in cholera cases [8]. There is also a surge in meningitis cases as the dry season begins [9]. Drivers for these are multiple and complex and need to be understood to design response strategies. There is hardly a quiet period for infectious disease epidemiologists and other public health professionals with several associated opportunities to learn lessons. As past and current leaders of NCDC, we consider capacity building to learn from our work through theoretical, applied, and operational research to be one of our most urgent priorities.

**Fig 1:**
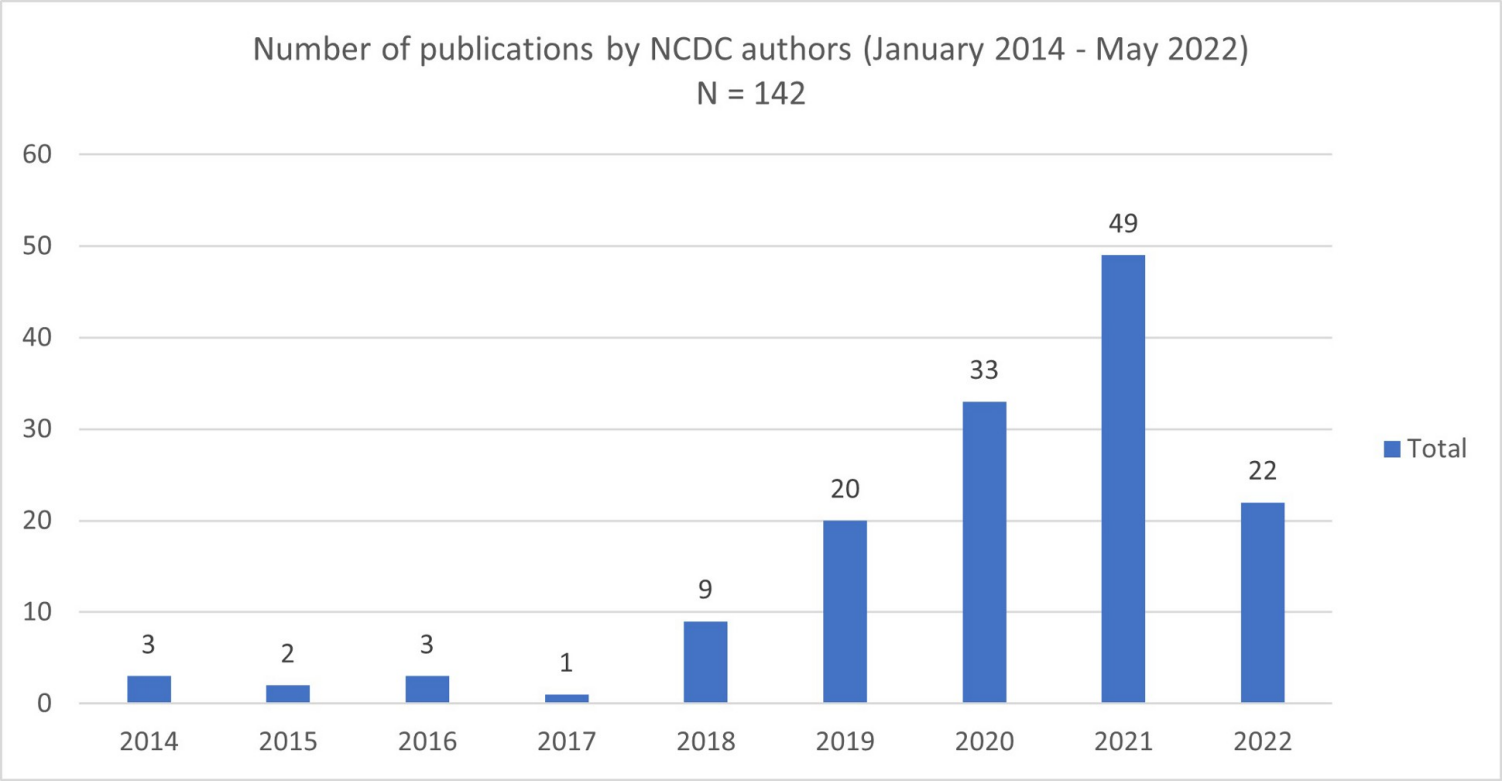


In 2016, NCDC-affiliated authors had fewer than five publications on the database PubMed (***Fig 1***), and there were very few others on the prevalent infectious disease in our context with Nigerian authors. However, there were many more publications by authors, mostly from high-income countries, on the work done in Nigeria without Nigerian authors who must have contributed significantly to the research before publication, and especially those in government institutions. This is a practice described as parachute research [10]. In addition to the immediately obvious impact, this practice means that these researchers are often recognised globally as experts for these infectious diseases and conditions, to the exclusion of local researchers. They often win awards, get grant funding and conference speaking opportunities which perpetuates this narration of exclusive expertise. Their views are used to shape global health discourse, developing recommendations that are often not suitable for the countries or communities where they should be implemented.

Despite these headwinds, through our work in NCDC, there are a few interventions that have enabled us to change this situation to some extent between 2016 and 2022– five of which we reflect on here.

Firstly, we re-introduced the development of several reports including daily and weekly outbreak situation reports, weekly epidemiological reports, and outbreak investigation reports amongst others. In addition to the obvious value of collecting and reporting data regularly to make decisions, these reports served as the foundation for the new manuscripts. In a context of limited resources where national public health agencies in Africa do not have professional scientific writers employed in their institutions unlike our European or American counterparts, the writing of reports by the people participating in outbreak response or activities, was a time consuming but necessary important foundation for the subsequent development of manuscripts.

Secondly, we prioritised the development of peer-review papers as part of the Nigeria Field Epidemiology Training Programme [NFETP]. Residents of the programme are often deployed as part of NCDC’s Rapid Response Teams during outbreaks. One requirement as part of their programme is the development and publication of a manuscript in a peer-reviewed journal. So, for every deployment, residents were encouraged to think about documenting lessons and sharing them while maintaining the required scientific requirements. This not only helped to develop their capacity but promoted the sharing of lessons in Nigeria’s health security, written by Nigerians.

Thirdly, we developed and introduced a mantra of “nothing for us without us” to all our partners. The NCDC often received support from international partners in the response to disease outbreaks, for which we are grateful. In some cases, these partners were deployed to work with us in-country. For every partnership at NCDC, we insisted that the partners could not use the data collected from our work together, for any reason, without prior written consent. It was not also accepted to develop a manuscript and to send us a final version for review. We requested that our partners worked with us, collaboratively in the development of manuscripts, and insisted that an NCDC author must always be first or last author for ownership, while encouraging partners to take on supporting roles. While this seemed like an important compromise at the beginning, it has become a norm, allowing for mutual respect and trust across various parties. Our more progressive partners were content with NCDC authors taking both first and last authorship as long as they contributed as appropriate for those positions.

Fourthly, in 2018, we established a research, training, and knowledge management unit at NCDC. In most Nigerian government institutions, there is a Department of Planning, Research and Statistics. However, we found that the existing structure did not meet our needs. While NCDC does not have the capacity to hire scientific writers yet, we established this unit to create a structure to promote and support research including scientific writing. This includes training staff on writing, creating standard operating procedures for data collection and use within the agency, developing a process for organisational approval of manuscripts, developing research collaborations with partners [for example, the Nigeria Cochrane Centre] amongst others.

Finally, we leveraged on the strength of NCDC’s young staff cadre hungry for professional growth. We encouraged members of staff to view the development of publications as an important benefit of their careers at NCDC. This meant they proactively identified issues to write about and put in efforts into this. Older colleagues often served as mentors, reviewing drafts, and contributing themselves. We also created a structure to prevent exploitation and several times, young people despite their level of seniority in the agency have led as first authors on significant papers.

While these five reflections are not exhaustive, they have seen NCDC grow from having less than 10 publications in 2016 to over 140 by April 2022 as indexed on PubMed (***Fig 1***). Of these, NCDC staff were first author for 33 publications and last author for 46 publications. In addition to these peer-reviewed publications, for every year between 2016 and 2020, NCDC staff have led the development of annual reports, weekly epidemiological reports, weekly situation reports during outbreaks, guidelines and monthly situation reports for epidemic-prone diseases in Nigeria [11, 12]. These have proven extremely useful for global health discourse. For example, NCDC’s monthly situation reports on monkeypox have been useful to several institutions around the world currently recording an unusual number of cases. By doing this, we are progressively enabling NCDC staff to be recognised as credible voices to tell our own stories and contributions to global health.

There are several other interventions that have enabled NCDC to lead on telling its own stories. The number of staff at NCDC increased from less than 100 in 2016 to over 500 as of January 2022. Of this, about 60% are non-administrative staff and are provided with opportunities to contribute to scientific publications. In negotiating support for NCDC, we included scientific writing development as a priority. The UK Health Security Agency, International Association of National Public Health Institutes [IANPHI], Africa CDC and African Journal of Laboratory Medicine have supported this at NCDC. In addition, journals have offered discounts and, in some cases, full waivers to NCDC papers. We intend to build on these interventions and new approaches, to increase the number of publications led by NCDC authors.

There are several imbalances in global health that perpetuate the inequalities in the authorship of publications, and we must continue to tackle these. However, there is also a role for governments and institutions in LMICs. We hope that other countries in Africa with similar challenges will consider adapting some lessons from our journey at NCDC.
